# Recent Advances in Neuroblastoma Research

**DOI:** 10.3390/cancers16040812

**Published:** 2024-02-17

**Authors:** John Inge Johnsen, Per Kogner

**Affiliations:** Childhood Cancer Research Unit, Department of Women’s and Children’s Health, Karolinska Institutet, 17177 Stockholm, Sweden; per.kogner@ki.se

Neuroblastoma is a neural crest-derived tumor of the peripheral nervous system that is a leading cause of cancer-related deaths in children. High-risk neuroblastoma often presents with metastasis at diagnosis and frequently acquires drug resistance [1]. Neuroblastomas display heterogeneous clinical behavior through chromosomal aberrations, where the gene amplification of *MYCN*, deletion of 1p or 11q and segmental gain of chromosome (Chr) 17q are associated with poor prognosis [1].

Neuroblastoma is an embryonal tumor, and the malignant mechanisms are shaped by its developmental origin on the neural crest. Recent detailed insights into the fetal development of the human adrenal gland have extended our knowledge of the cellular identities and possible differentiation trajectories of neuroblastoma [2,3]. However, it is still not understood how this embryonic plasticity relates to the origin of neuroblastomas. In vitro, it has been shown that neuroblastoma cell lines can occur with two distinct phenotypes: the adrenergic (tADR) and mesenchymal (tMES). Both phenotypes can be distinguished by their transcriptional and epigenetic characteristics, but they are genetically identical [4]. While different clinical phenotypes have been discovered in vivo based on comprehensive RNA expression analyses or their epigenetic subtypes, the main aspects of tumor plasticity, cellular interconversions and the origin of neuroblastoma are still undetermined [2,5]. This is of great clinical relevance, as molecular heterogeneity and cellular plasticity are directly connected to the primary clinical challenges of the disease, such as metastatic spread, therapy resistance and clinical progression or relapse. Despite the development of risk-based, complex, intensified, multimodal therapy, neuroblastoma survival is still unsatisfactory in children with high-risk disease [6]. Hence, in order to develop improved therapies for better survival, fewer side effects and, ultimately, a better quality of life for children with neuroblastoma, it is imperative to identify new treatment options. These may be found through establishing the cellular origin, mechanisms of drug resistance and druggable targets of neuroblastoma, which can be translated into clinical trials. In this Special Issue, different facets of neuroblastoma biology, including investigations into the potential founder cells of different neuroblastoma subtypes, the vascularity of tumor tissues as a tool for identifying viable tissues in chemo-treated tumors, preclinical neuroblastoma models of relevance for the development of new treatment options, and investigations into new compounds for the treatment of neuroblastoma, are described and discussed.

The transcription factor MYCN is a hallmark of high-risk neuroblastoma, and 20–30% of patients present with tumors that demonstrate an amplification of the MYCN gene at diagnosis. In this Special Issue, Bartolucci et al. [7] present a detailed description of MYCN as a prognostic indicator of high-risk neuroblastoma and a driver of neuroblastoma development through its ability to disturb key processes during neural differentiation. This leads to continued proliferation, metabolic changes, an inhibition of apoptotic processes and a remodeling of the external environment to support tumor growth. MYCN represents a highly relevant molecule for targeted therapy, given the fact that high expression is almost exclusively present in tumor cells, while limited or transient expression is observed in normal cells and tissues. Even more importantly, in neuroblastoma, *MYCN*’s amplification and high expression are correlated with rapid tumor cell proliferation, tumor progression and poor patient prognosis. However, despite numerous attempts to establish one over the last three decades, no efficient inhibitor of MYCN is available in the clinic. This has been attributed to the lack of specific drug interaction sites within the MYCN molecule and the fact that transcription factors have, in general, been difficult to target precisely. Therefore, alternative strategies to inhibit the effects of MYCN in high-risk neuroblastoma are being investigated. These include the targeting of MYCN transcription using bromodomain inhibitors and inhibitors of the Mdm2-Tp53 axis, or compounds affecting MYCN protein stability, of which the most prominent are small molecule inhibitors of PI3K/mTOR signaling and Aurora-A kinase.

It has long been known that neuroblastomas that occur in the adrenal gland differ fundamentally from ganglionic neuroblastomas. Adrenal tumors are more aggressive and have a worse prognosis than ganglionic tumors. Genetically, adrenal neuroblastomas frequently have segmental chromosomal aberrations, including *MYCN* amplification, while ganglionic tumors usually present with numerical chromosomal aberrations. Therefore, it has been assumed that adrenal and ganglionic neuroblastoma could be initiated by different founder cells during embryonal development. This subject is investigated by Sriha et al. [8], who use cultures of mouse-sympathetic neuroblasts and chromaffin cells to investigate whether different tumor properties can be linked to specific tumor initiator cells in the adrenals or sympathetic ganglia. They established cell cultures of chromaffin cells derived from postnatal mouse adrenals and neuroblasts from the paravertebral ganglia to study the signaling pathways that are important for proliferation. No differences in proliferation were found when using inhibitors of molecules known to be important for the development of sympathoadrenal cells or neuroblastoma tumorigenesis, such as WNT, IGF1, ALK and EZH2/PRC2. However, BET inhibition selectively suppressed the proliferation of chromaffin cells and CDK7/12/13 inhibition suppressed the growth of neuroblasts. The findings by Sriha et al. support the hypothesis that these two neuroblastoma types derive from different founder cells.

High-risk neuroblastoma continues to be a clinical challenge, with poor survival despite intensive multimodal therapies. Therefore, alternative treatment options are urgently needed. Two reports in this issue, by Wang et al. and Ahmed et al. [9,10], describe the effects of natural compounds, cannabinoids deriving from *Cannabis Sativa* L. and marine cyanobacterial peptides, respectively, on neuroblastoma growth. Cannabinoids have demonstrated anticancer effects in preclinical in vitro and in vivo models, and several clinical trials on different synthetic or natural cannabinoids are ongoing for adult cancers [11]. However, there are also reports that cannabinoids have enhancing effects on the growth of cancer cells [11]. Wang et al. show that the phytocannabinoid cannabinol attenuates cell proliferation, angiogenesis and invasion by inhibiting the activity of PI3K/AKT signaling and upregulating miR-34a, which directly silences E2F1 and guides the production of rRiMetF31, which targets 6-phosphofructo-2-kinase/fructos-2,6-biphosphate-3 (PFKFB3) in neuroblastoma cells [9]. Several studies have reported that inhibiting key molecules in the PI3K/AKT/mTOR signaling pathway suppresses the growth of neuroblastoma in preclinical models, and mTOR inhibitors are currently undergoing clinical testing and are in clinical use for patients with relapsed disease [1]. Increased PFKFB3 activity has been observed in cancer cells, as well as highly proliferative normal cells, resulting in increased glycolysis [12]. In neuroblastoma, a high expression of PFKFB3 correlates with poor survival [13]. Hence, as the study by Wang et al. indicates, cannabinoids have suppressing effects on neuroblastoma growth and may represent an alternative treatment option that merits further testing in animal models [9].

Ahmed et al. [10] give an overview of the reported anticancer activities of marine peptides isolated from cyanobacteria, with a particular focus on their effects on neuroblastomas. Several cyanobacterial peptides derived from different sources have been tested and shown to have growth-inhibiting effects on neuroblastoma cells. Their mechanisms of action are diverse, including the induction of apoptosis, cell cycle arrest, autophagy and the blocking of sodium channels. However, the short half-life of these peptides has hindered more detailed in vivo studies and further research is needed to develop more stable variants of these peptides in order to determine their potential as anti-neuroblastoma drugs.

Discriminating between malignant, necrotic and healthy tissues is a challenge for surgical oncology. In neuroblastoma, tumor vascularity differs considerably between prognostically favorable and unfavorable cases [14]. However, identifying particular microvascular traits has not been thoroughly analyzed as a potential diagnostic for differentiating between different histopathological groups and their responses to therapy. Privitera et al. used scanned histopathology slides to examine the variation of vascular density within different areas of neuroblastoma tumors, and combined this with photoacoustic imaging, which provided high-resolution 3D images of the tumor tissues. Using this approach, they were able to show that viable tumor tissues from neuroblastoma patients are associated with higher microvascular densities after chemotherapy, compared to poorly differentiated neuroblastomas [15]. These findings suggest that photoacoustic imaging combined with histopathologic scanning can be used to identify different vasculature signatures, which can act as reliable markers for distinguishing between viable and non-viable tissues in chemotherapy-treated tumors.

Preclinical in vitro and in vivo models are central to cancer research as tools for analyzing the effects of drugs and vaccines on tumor growth, studying tumor development, evaluating disease mechanisms and investigating specific genomic aberrations and the biochemical pathways that are dysregulated in cancer. For neuroblastoma, which is a relatively rare tumor with a high degree of heterogeneity, the availability of relevant patient-derived preclinical models is of the utmost importance, as therapy evaluation and the screening of potential new treatment options are limited. In this issue, Krawczyk and Kitlinska present an extensive update on the current status of preclinical models for neuroblastoma research [16]. Compared to 2D cultures, 3D cultures, or more specifically patient-derived organoid or tumoroid cultures, offer great promise as pre-clinical cancer models to improve drug development, as they closely resemble the heterogeneous genetic and morphological composition of cancer cells in the original tumor. Although patient-derived tumoroids have been established from cancers of diverse origins which closely resemble the original tumor, the establishment of organoid cultures from neuroblastoma patient tissues directly has been challenging [17]. However, patient-derived xenograft models of neuroblastoma in immunocompromised mice, which mimic the features of the original tumors, have been established and successfully used to monitor drug efficacy [18].

Genetically engineered mouse models (GEMMs) offer an excellent tool for studying the effects of specific genes on tumorigenesis. For neuroblastoma, the TH-MYCN and modified models thereof have been extensively used for studying the effects of MYCN on neuroblastoma development and testing different novel treatments. Additionally, GEMMs of mutated *ALK*, as well as *IGF2BP1* [19], induce neuroblastoma, although with a lower tumor penetrance and/or prolonged time for tumor development compared to the TH-MYCN model. However, none of the described GEMMs of neuroblastoma exhibit frequent metastatic tumors in a manner that recapitulates patient tumors. Hence, there is currently a lack of reliable preclinical models that recapitulate the metastatic spread observed in children with neuroblastoma. Developing relevant preclinical metastatic models that recapitulate human diseases should be a priority, as the majority of patients with neuroblastoma succumb to metastatic, recurrent or relapsed disease.

This Special Issue “Recent Advances in Neuroblastoma Research” is a glimpse of the current research being carried out within the field of neuroblastoma. Future research should be focused on resolving the issue of neuroblastoma heterogeneity and its mechanisms of treatment resistance, establishing preclinical models that mimic metastatic disease and developing efficient treatments for patients with metastatic, recurrent or relapsed disease, for whom we currently have no efficient treatment options. The chances of survival for patients with refractory or relapsed neuroblastoma is currently dismal. However, there are several ongoing clinical trials focusing on new modes of treatment for this group of patients (https://clinicaltrials.gov/search?cond=neuroblastoma%20relapse, access on 2 February 2024). Furthermore, recent detailed mapping of the immune cell landscape of neuroblastoma and novel immunotherapy approaches, including GD2-CAR-T and ALK-CAR-T, have indicated that there are new approaches to treating and potentially curing patients with refractory or relapsed neuroblastoma on the horizon [20,21,22,23].
